# Gastrointestinal delivery of propofol from fospropofol: its bioavailability and activity in rodents and human volunteers

**DOI:** 10.1186/s12967-015-0526-9

**Published:** 2015-05-29

**Authors:** Krystyna M Wozniak, James J Vornov, Bipin M Mistry, Ying Wu, Rana Rais, Barbara S Slusher

**Affiliations:** Eisai Inc., Baltimore, MD USA; Johns Hopkins Drug Discovery, Johns Hopkins School of Medicine, The John G. Rangos, Sr. Building, 855 N. Wolfe Street, Baltimore, MD 21205 USA; Department of Neurology, Johns Hopkins School of Medicine, Baltimore, MD USA; Department of Psychiatry, and Neuroscience, Johns Hopkins School of Medicine, Baltimore, MD USA; Medpace, Cincinnati, OH USA; Center for Veterinary Medicine, FDA, Derwood, MD USA

**Keywords:** Propofol, Prodrug, Non-IV, Pain, Hyperalgesia

## Abstract

**Background:**

Propofol is a safe and widely used intravenous anesthetic agent, for which additional clinical uses including treatment of migraine, nausea, pain and anxiety have been proposed (Vasileiou et al. Eur J Pharmacol 605:1–8, 2009). However, propofol suffers from several disadvantages as a therapeutic outside anesthesia including its limited aqueous solubility and negligible oral bioavailability. The purpose of the studies described here was to evaluate, in both animals and human volunteers, whether fospropofol (a water soluble phosphate ester prodrug of propofol) would provide higher propofol bioavailability through non-intravenous routes.

**Methods:**

Fospropofol was administered via intravenous, oral and intraduodenal routes to rats. Pharmacokinetic and pharmacodynamic parameters were then evaluated. Based on the promising animal data we subsequently conducted an oral and intraduodenal pharmacokinetic/pharmacodynamic study in human volunteers.

**Results:**

In rats, bioavailability of propofol from fospropofol delivered orally was found to be appreciable, in the order of around 20–70%, depending on dose. Availability was especially marked following fospropofol administration via the intraduodenal route, where bioavailability approximated 100%. Fospropofol itself was not appreciably bioavailable when administered by any route except for intravenous. Pharmacologic effect following oral fospropofol was confirmed by observation of sedation and alleviation of thermal hyperalgesia in the rat chronic constrictive injury model of neuropathic pain. The human data also showed systemic availability of propofol from fospropofol administration via oral routes, a hereto novel finding. Assessment of sedation in human volunteers was correlated with pharmacokinetic measurements.

**Conclusions:**

These data suggest potential utility of oral administration of fospropofol for various therapeutic indications previously considered for propofol.

**Electronic supplementary material:**

The online version of this article (doi:10.1186/s12967-015-0526-9) contains supplementary material, which is available to authorized users.

## Background

Propofol (2, 6-diisopropylphenol) is an intravenous short-acting anesthetic agent that has gained wide acceptance for inducing and maintaining anesthesia and for procedural sedation. Animal and clinical data suggest that propofol has a variety of non-hypnotic effects that may have therapeutic application at non-sedative doses [1, 2]. Propofol also has diverse pharmacology that might prove useful in several conditions, including prolongation of inhibitory postsynaptic currents mediated by GABA A receptors [3] as well as enhancing GABA release via presynaptic mechanisms [4].

Potential clinical utility for propofol has been reported in various conditions including anxiety [5, 6], migraine [7–10], analgesia [11, 12], emesis [13, 14] and pruritis [15–17], all at exposures below those causing sedation.

In spite of this potentially useful and unique pharmacology, the clinical use of propofol in other therapeutic areas has been limited by its formulation as a short-acting intravenous emulsion. Propofol’s insolubility in water requires its formulation in a lipid emulsion [18] using complicated manufacturing processes with ensuing limited storage time because of the risk of microbial contamination [19]. Propofol is not orally bioavailable in animals or in humans [20, 21] possibly due to limited aqueous solubility, and first pass metabolism by the liver. It has been reported that intravenous administration of the lipid emulsion undergoes an extraction of 80% by the liver in animals [22–24] and in humans [25, 26].

Fospropofol (Lucedra™) is a water-soluble, phosphono-*O*-methyl prodrug of propofol that was approved as an alternative to propofol for monitored anesthesia care during procedures in the United States [19, 27]. The aqueous solubility of fospropofol allows it to be formulated for intravenous use without the oil in water emulsion formulation required for propofol. Fospropofol is rapidly metabolized by endothelial alkaline phosphatases to release propofol, phosphate and formaldehyde. Formaldehyde is rapidly converted to formate and safely eliminated, similar to the other available phosphate methyl prodrugs such as fosphenytoin. Sedative effects appear to be due entirely to the propofol liberated from the prodrug. However, prodrug metabolism leads to differences from propofol in its onset, peak effects and duration of action [19, 28, 29]. Fospropofol was generally well tolerated in clinical trials with only mild to moderate adverse events reports, mostly transient in nature [30]. Thus, as an intravenous sedative, fospropofol has several advantages over propofol, including less pain at site of injection, less potential for hyperlipidemia with long-term use and less chance of bacteremia in patients.

We hypothesized that the oral bioavailability of propofol from fospropofol might differ from that of propofol due to its novel properties of water solubility and lack of emulsion formulation. In the animal and clinical studies reported here we demonstrate, for the first time, the successful delivery of propofol by administration of the prodrug fosprofol through the gastrointestinal tract, a property that may ultimately be exploited for clinical use.

## Methods

### Animal studies, drugs and formulations

Male Sprague–Dawley rats (Charles River, MD, USA) weighing 200–250 g were used, unless otherwise noted. Animals were housed in groups of three inch suspended polycarbonate cages (18 inch long, 9 inch wide and 9 inch high) under a 12 h light/dark cycle. Food (Harlan/Teklab) and water (filtered and delivered via an automatic watering system) were provided ad libitum. All procedures were conducted in compliance with the laws, regulations and guidelines of the National Institutes of Health (NIH/PHS) and with approval from the local Animal Care and Use committee.

For animal studies, fospropofol dosing solution was made by dissolving powder in water (for oral and intraduodenal administration) or saline (for intravenous administration) into an administration volume of 1 or 2 mL/kg, as noted.

#### Pharmacokinetic studies in rats

Male Sprague–Dawley rats (225–250 g) underwent implantation with indwelling jugular vein catheters for plasma sampling. Animals receiving intravenous (IV) drug administration also underwent femoral vein catheterization and, after full recovery were attached to an electronic infusion pump and administered vehicle or various concentrations of fospropofol in 1 mL total volume by gradual constant rate infusion over 10 min. Intraduodenal (ID) administration was via previously implanted catheters (HILLTOP Labs, PA) in a constant volume of 2 mL/kg body weight, by slow infusion. Oral administration (PO) was performed via a curved bulb-tipped feeding gavage tube attached to a syringe inserted carefully through the oesophagus via the side of the mouth of manually restrained rats using an administration volume of 1 mL/kg. On the day of testing, control blood samples were taken from the jugular vein prior to dosing of test compounds in conscious rats. In these studies, intravenous doses were based on previously reported behavioral and pharmacokinetic studies with fospropofol. The PO and ID doses were established based on preliminary experiments with behavioral observation, using higher doses based on lower expected oral bioavailability.

After fospropofol administration, blood samples (0.5 mL) were taken at 5, 15, 30, 45, 60, 120, 240 and 360 min post dose. An equivalent volume of blood taken from donor rats of the same strain was administered after each blood sample withdrawal, in an effort to maintain blood volume as previously described [31–33]. Approximately 0.05 mL of 200 mg/mL of sodium orthovanadate (SOV) solution was added to the heparinized blood collection tubes prior to blood collection to prevent ex vivo conversion via alkaline phosphatases (ALP). The blood samples were mixed, cooled and subsequently centrifuged at 3,000 rpm and 4°C for 10 min within 30 min of collection. Plasma samples were stored at −20°C until analysis.

#### Sedative studies in rats

The relative potency of fospropofol given via different administration routes was investigated in rats, using sedation as an end point. Intravenous fospropofol doses of 5–40 mg/kg were chosen based on previous studies [34]. Based on expected lower oral bioavailability, doses of 100–400 mg/kg were chosen for the PO and ID studies. Following administration of fospropofol, two experimental observers blinded to the treatment, graded the behavior of the rats (n = 2–3 per experimental group) every 5 min for a total of 120 min after administration. The scoring system was on a 0–4 scale where 0 = alert and completely responsive, 1 = alert but less active and ‘wobbly’, 2 = awake but drowsy with periods of in-activity or mild sedation, 3 = inactive but readily arousable or moderately sedated, 4 = unresponsive, unconscious or deeply sedated. Scores were averaged across treatment groups per time point.

#### Neuropathic pain studies in rats

This study was performed as previously described by our group [35]. In brief, male Sprague–Dawley rats (200–250 g) were anesthetized with halothane. The common sciatic nerve on one hind limb was exposed by separating the biceps femoris from the gluteus superficialis. The nerve was subsequently isolated from the surrounding tissue and four ligatures (4.0 chromic gut) were tied loosely around it with about 1 mm spacing. On the other hind limb of the rat, the nerve was similarly isolated but no ligatures were placed, (sham surgery). Thermal pain sensitivity was evaluated using a plantar test apparatus according to previous methods [36]. In brief, this involved applying a constant infrared stimulus to the plantar surface of the hind paw using a Basile Plantar apparatus (Ugo Basile, Vaarese, Italy). Withdrawal latency was measured as the time taken for the rat to withdraw its paw from the heat source to the nearest 0.1 s. The “difference score” was calculated by subtracting the average latency of the non-ligated versus ligated side. Animals were habituated to the test chambers (clear plastic compartment maintained in a quiet room) for several hours over 3–4 days, prior to any measurement. Baseline hyperalgesia was recorded 10–12 days post-surgery after the habituation. On test day, each animal received either fospropofol (50, 75 or 100 mg/kg) or vehicle (distilled water) via oral gavage in a volume of 2 mL/kg in a randomized and blinded fashion. Withdrawal latency measurements were then recorded five times for both the operated and sham hind paws of each rat starting from 45 to 60 min post dose. The final latency measurement represents a mean of the last four out of a total of five responses, each being taken at least 5 min apart on the same paw. The difference in response latency for each rat for each leg was calculated and used to determine the mean latency difference response time for each group.

### Human studies, drugs and formulations

Human studies were approved by the institutional review boards at PRA Health Sciences in Groningen, Netherlands and were conducted in accordance with the ethical principles that have their origin in the Declaration of Helsinki and the International Conference on Harmonization guideline E6: Good Clinical Practice. All participants provided written, informed consent before study entry and had the right to withdraw from the study at any time.

In human study 1, Fospropofol disodium was formulated as a sterile aqueous solution at a concentration of 20 mg/mL. Each vial provided contained 20 mL of solution, suitable for intravenous injection. Fospropofol was administered as a single dose of 400 mg orally, directly into the duodenum by gastroscopy or intravenously over 10 min. In human study 2, fospropofol disodium in capsules (200 mg) or matching placebo was administered orally.

#### Fospropofol studies in human volunteers

The first study was an absolute bioavailability study of fospropofol conducted at a single center (PRA Health Sciences) in Groningen, Netherlands, as a three-way crossover study. The study enrolled 7 healthy male volunteers between 18 and 45 years of age inclusive, with a body mass index between 18 and 28 kg/m^2^. Subjects stayed in the clinical unit for three consecutive periods of 3 days each, with a 3-day washout, between periods. For six subjects, the order of the administration routes was as follows—period 1: PO, period 2: ID, and period 3: IV. For one subject the order of the administration routes was the following—period 1: ID, period 2: IV and period 3: PO. In this study a single dose of 400 mg was administered by each route. Blinding and placebo control was impractical due to the requirement of unsedated endoscopic administration of fospropofol into the duodenum. Safety and overall pharmacodynamic effect was evaluated based on adverse events, vital signs, electrocardiogram (ECG), Modified Observer’s Assessment of Alertness/Sedation (OAA/S) score, clinical laboratory tests, and physical examination.

A second single ascending dose study was subsequently performed to assess the safety, tolerability, and pharmacokinetics of oral administration of fospropofol as a capsule. This was a double-blind, randomized, crossover, placebo-controlled, single ascending dose study. Ten healthy volunteers were enrolled, 6 males and 4 females between 18 and 45 years of age inclusive with a body mass index between 18 and 28 kg/m^2^. Each subject received four ascending oral doses of fospropofol disodium (200, 600, 1,000 and 1,200 mg) and one of placebo. Placebo was administered randomly, in one of the five periods. Subjects stayed in the clinical research unit over 3 days per treatment for five consecutive treatments. Between treatments, there were wash-out periods of at least 6 days, during which interim safety evaluations were made to assess the safety of the subsequent higher doses.

Fopropofol disodium in capsules (200 mg) or matching placebo was administered orally. Pharmacokinetic parameters, safety and pharmacodynamic effect were assessed in a manner similar to that in Study 1 described above. In addition, the digit symbol substitution test (DSST) and BIS Index (a commercially available EEG derived measure of anesthesia and sedation, Coviden, Mansfield, MA, USA) were added as additional pharmacodynamics measures.

In the first study, 6 mL blood samples were collected following the PO and ID fospropofol treatment periods at times of pre-dose, 5, 10, 20, 30, 45, 60, 90 min and 2, 4, 6 and 9 h post-dose and in the IV fospropofol treatment period at pre-dose and at 5, 10, 15, 20, 30, 45, 60 and 120 min post-dose. In the second study a 6 mL blood sample was collected following each fospropofol PO dose (200, 600, 1,000 and 1,200 mg) at pre-dose, 5, 10, 20, 30, 45, 60, 90 min and 2, 4, 6 and 9 h post-dose. The blood was collected in a sodium heparin vacutainer tube containing 60 mg SOV, inverted approximately eight times to dissolve SOV and placed on dry ice until it was centrifuged at 3,000 rpm for 10 min at 4°C to harvest plasma. Plasma samples were then stored at −20°C until analysis.

### Bioanalysis of propofol and fospropofol

Fospropofol and propofol in human and rat plasma were quantified using a validated high performance liquid chromatography (HPLC) with a tandem mass spectrometry method (LC/MS/MS) and an HPLC fluorescence detection method respectively, as described below.

For fospropofol analysis, fospropofol-d^6^ (internal standard prepared in 1.0 M ammonium acetate buffer) was added to the plasma samples (rat or human: 0.05 mL) and subsequently extracted using a solid phase extraction (SPE). The SPE cartridges were conditioned by gravity with methanol and 1.0 M ammonium acetate solution in water. The plasma samples with internal standard were loaded on the cartridges, washed with water and 10% methanol/water and eluted with methanol. The tubes were then evaporated under nitrogen and the residues reconstituted with 50/50 methanol/25 mM ammonium acetate in deionized water. The sample extract was then injected onto a reversed phase HPLC Zorbax Eclipse XDB-C18 column. The separated analytes were detected using tandem mass spectrometry (MS/MS) detection. Fospropofol was quantitated by peak area ratio to its internal standard by mass spectrometry using a selective reaction monitoring mode (for fospropofol m/z = 287.1 → 79.1, and for D_6_-fospropofol m/z = 293.1 → 79.1). The assays were linear with correlation coefficient of (R^2^) >0.99 over the range of 10–2,000 ng/mL for rat plasma and 5–1,000 ng/mL for human plasma. Fospropofol was stable for 98 days at −20°C in rat plasma and 464 days at −20°C in human plasma.

The propofol plasma assay method was modified from an earlier published method by Plumb et al. [37]. In brief, 4-[tert-octyl] phenol, (internal standard) was added to the plasma samples (0.05 mL for rat or 0.2 mL for human) and extracted using a 3M Empore C-18-SD 4 mm SPE cartridge (Millipore, Billerica, MA, USA). A mixture of plasma sample with drug and internal standard in ammonium acetate buffer was passed through the SPE conditioned with methanol and water. The SPE cartridge was further subjected to three wash steps; first with 1 mL of water, second with 1 mL of 10% methanol in water, and third with 20% acetonitrile in water. Finally the analytes were eluted using two times 0.15 mL of acetonitrile. The final eluants (~0.3 mL) were diluted with 0.3 mL of water, and injected onto an HPLC system equipped with a C-18 analytical column (5 µm, 150 × 3.9 mm) and fluorescence detector set at excitation and emission wave lengths of 275 and 310 nm, respectively. Propofol was quantitated by peak height ratio to internal standard. The assays were linear with correlation coefficient of (R^2^) >0.99 over the range of 5–2,000 ng/mL for both rat and human plasma. As reported by Shah et al. [38], the precise measurement of plasma propofol using this method may be compromised under conditions of severe hemolysis, as this causes insolubility of the added SOV during sample collection which could result in incomplete ALP inhibition. Given this, hemolysis was avoided or minimized in both the preclinical and clinical studies by conducting sample processing in a cold environment. Propofol was found to be stable at −20°C for 65 days in rat plasma and 347 days in human plasma. All rat and human study samples in this study were analyzed without exceeding the stability sample integrity during validation of the methods.

### Pharmacokinetic parameter calculations

Pharmacokinetic parameters were determined for fospropofol and propofol from plasma including area under the concentration–time curve from time of dosing to the last measured concentration (AUC_(0-t)_), peak concentration (C_max_), time to reach maximum concentration (T_max_), terminal phase half-life (t_1/2_) and area under the concentration–time curve from time of dosing to infinity (AUC_(0−∞)_). Absolute bioavailability (F) was calculated as the ratio of AUC_(0−∞)_ resulting from PO or ID administration to AUC_(0−∞)_ following IV administration, correcting for the specific doses used. The parameters were summarized using descriptive statistics.

## Results

### Bioavailability of fospropofol and propofol following IV, PO and ID dosing of fospropofol in rats

**Figure 1:**
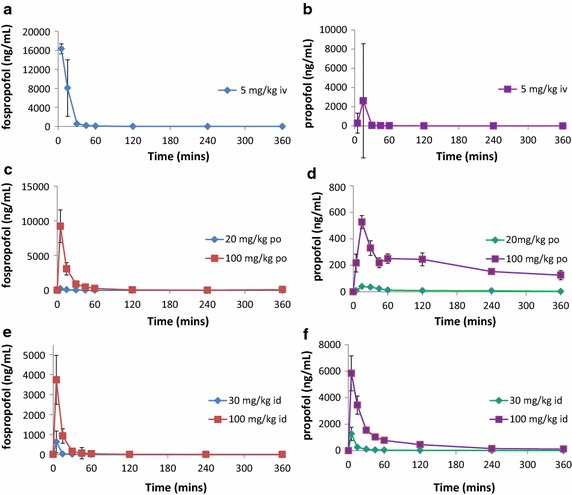
Plasma-concentration-time curves of fospropofol and propofol after intravenous, oral and intraduodenal administration of fospropofol to rats. Intravenous administration of fospropofol (5 mg/kg) resulted in the expected immediate high concentrations of both fospropofol (**a**) and propofol (**b**). Oral administration of higher doses of fospropofol (20 or 100 mg/kg) resulted in lower peak fospropofol plasma exposure (**c**) but significant and prolonged propofol exposure (**d**). Intraduodenal administration of high doses (30 or 100 mg/kg) resulted in similar fospropofol levels in the plasma (**e**) but relatively higher peak propofol exposure (**f**). Data shown as mean ± SEM.

Following IV, PO and ID administrations the C_max_ and AUC of fospropofol increased with dose (Table 1; Figure 1a–d). However, the increase in both parameters was less than dose proportional following IV administration and greater than dose proportional following both PO and ID administrations. The absolute bioavailability of fospropofol was low following both PO and ID administrations, ranging between 0.448 and 3.46% (PO 20 and 100 mg/kg) and 0.264 and 1.03% (ID 30 and 100 mg/kg), respectively.

**Table 1:** Mean (±SD) pharmacokinetic parameters of fospropofol following fospropofol administration in rats

Route	Dose (mg/kg)	C_max_ (µg/mL)	AUC_(0−t)_ (µg h/mL)	AUC_0−∞_ (µg h/mL)	T_1/2_ (h)	V_d_ (L/kg)	CL_p_ (L/h/kg)	F^a^ (%)
IV	5	16.3 (±1.80)	3.15 (±0.29)	3.15 (±0.28)	0.49 (±0.28)	1.02 (±0.66)	1.38 (±0.125)	–
PO	20	0.23 (±0.07)	0.05 (±0.008)	0.06 (±0.007)	0.19 (±0.27)	–	–	0.448
PO	100	9.23 (±4.09)	2.16 (±0.99)	2.18 (±0.99)	0.49 (±0.24)	–	–	3.46
ID	30	0.17^b^	0.044	0.05	0.21nd	–	–	0.264
ID	100	3.74 (±2.12)	0.644 (±0.38)	0.65 (±0.39)	0.23 (±0.1)	–	–	1.03

*nd* not determined as only one rat showed terminal elimination.

^a^Ratio of mean of AUC(_0−∞_) to reference treatment of 5 mg/kg IV fospropofol.

^b^N = 1.

The C_max_ and AUC of propofol increased with dose. The increase in C_max_ and AUC was dose proportional for IV administration; in contrast, these parameters increased although not dose proportionally, following PO and ID administration (Table 2). The propofol bioavailability following fospropofol administration via the PO and ID routes ranged between 22.7 and 70.5% (PO 20 and 100 mg/kg fospropofol) and 47.3–141% (ID 30 and 100 mg/kg fospropofol), respectively.

**Table 2:** Mean (±SD) pharmacokinetic parameters of propofol following fospropofol administration in rats

Route	Dose (mg/kg)	C_max_ (µg/mL)	AUC_(0−t)_ (µg h/mL)	AUC_0−∞_(µg h/mL)	T_1/2_ (h)	F^a^ (%)
IV	5	0.29 (±0.04)	0.12 (±0.02)	0.14 (±0.03)	ND	ND
PO	20	0.04 (±0.001)	0.06 (±0.04)	0.13 (±0.12)	4.66 (±4.13)	22.7
PO	100	0.53 (±0.08)	1.21 (±0.2)	1.96 (±0.6)	4.13 (±1.12)	70.5
ID	30	1.27 (±0.87)	0.353 (±0.14)	0.398 (±0.14)	2.85 (±0.87)	47.3
ID	100	5.84 (±2.29)	3.57 (±0.57)	3.95 (±0.48)	2.32 (±0.73)	141

^a^Ratio of mean of AUC(_0−∞_) to reference treatment of 5 mg/kg IV fospropofol.

### Sedative effects of PO and ID administration of fospropofol in rats

**Figure 2:**
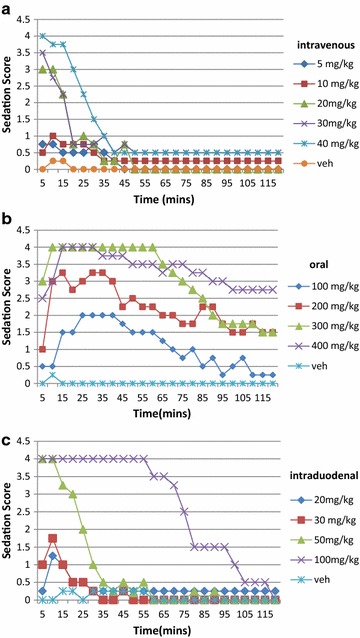
Sedation induced by fospropofol administered via **a** IV, **b** PO and **c** ID routes. The scoring system was on a 0–4 scale where 0 = alert and completely responsive, 1 = alert but less active and ‘wobbly’, 2 = awake but drowsy with periods of in-activity, 3 = generally sedated/inactive but readily arousable, 4 = unresponsive or unconscious. Sedative activity was assessed by blinded observers in 2–3 rats per treatment group.

Intravenous administration of fospropofol rapidly induced a dose-related sedation at 10–40 mg/kg (Figure 2a). The sedative effects were evident within 1 min and abated within 30 min after infusion.

After PO administration, animals displayed a rapid (within 5–10 min of dosing) dose-dependent onset of sedated behavior, followed quickly by loss of consciousness in the 300 and 400 mg/kg groups (Figure 2b), which lasted for up to approximately 1 h. Rats in the intermediate PO dose groups (100–200 mg/kg) displayed signs of mild to moderate sedation lasting about 1–2 h. In general, onset of sedation was slower and of longer duration after PO administration compared to the IV route (Figure 2a).

Similar to IV administration, ID fospropofol resulted in a similar rapid onset of sedation (within 5 min of its administration), followed by loss of consciousness in the higher dose groups. The onset of sedation was slightly faster than after PO administration and required lower doses (similar to those associated with the IV route) for the same maximal effect (Figure 2a, c). The duration of effect after ID administration was shorter than after PO administration at these lower doses, generally consistent with the time course predicted by pharmacokinetics.

### Analgesic effects of PO fospropofol administration in rats

**Figure 3:**
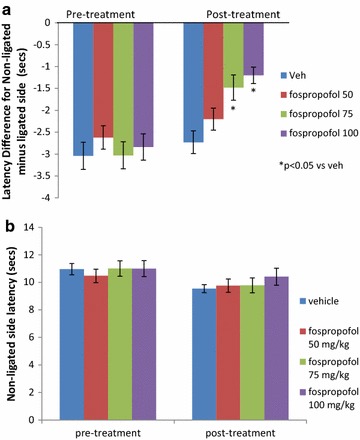
Analgesic effect of orally administered fospropool in rat chronic constrictive injury model of neuropathic pain. Withdrawal latency measurements were taken starting 45–60 min post-dose. Fospropofol was effective in reducing hyperalgesia at doses of 75 and 100 mg/kg (**a**). This effect was not due to a non-selective sedative effect as latency on the non-ligated side did not change with fospropofol treatment (**b**). n = 10 rats per group. p < 0.05 vs vehicle noted as “*”. Data shown as mean ± SEM.

Fospropofol was active in alleviating thermal hyperalgesia in the rat chronic constrictive injury model of neuropathic pain at doses of 75 and 100 mg/kg PO, but not at 50 mg/kg (Figure 3a). These effects were not due to general sedative effects as reflected by no change in latencies of response to stimuli on the sham (non-ligated side) in fospropofol treated rats vs vehicle (Figure 3b). In a separate study, rats (n = 10 per group) were dosed with fospropofol PO at 75 mg/kg in a volume of 2 mL/kg. Withdrawal latency was then tested at different time points following dosing (1, 2 and 4 h). Fospropofol was effective only when tested at 1 h after administration and not after longer time periods (data not shown). Mean absolute latencies of ipsilateral paw before and after fospropofol or vehicle treatment are shown in Additional file 1: Figure S1.

### Bioavailability of fospropofol and propofol in human volunteers following PO and ID fospropofol

**Figure 4:**
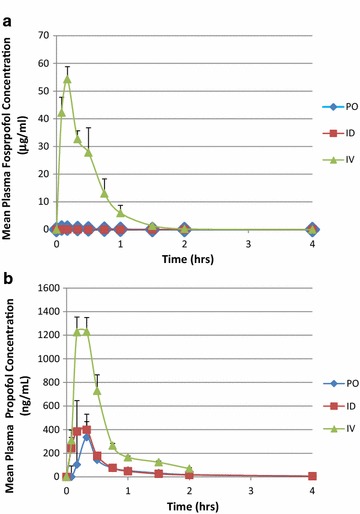
Pharmacokinetic profile of mean (±SEM) fospropofol and propofol concentrations in plasma following administration of fospropofol to human subjects by PO, IV and ID routes. A single dose of 400 mg was administered to seven volunteers in a sequential crossover design. Almost no plasma fospropofol was detected when administered by any non-intravenous route (**a**). In contrast, propofol bioavailability from fospropofol was substantial, ranging between 34 and 48% respectively by AUC (**b**). Data shown as mean ± SEM.

*Study 1* In this three way crossover study, absorption of fospropofol was rapid following PO and ID administrations, with T_max_ of 0.08 and 0.17 h, respectively. The mean plasma concentrations of fospropofol declined rapidly after reaching C_max_ (Figure 4a). Compared with IV fospropofol administration, the PO and ID fospropofol resulted in extremely low mean plasma concentrations of fospropofol. Compared with the C_max_ of IV fospropofol, the PO and ID C_max_ values were approximately 88- and 534-fold lower, respectively (data not shown). The absolute bioavailability of fospropofol after PO and ID administration was very low (1% for PO and 0.1% for ID administration). The mean t_1/2_ of fospropofol was similar following all methods of delivery (0.32, 0.28, and 0.28 h for PO, ID and IV, respectively).

Compared with the C_max_ of propofol after IV administration of fospropofol, the C_max_ values after PO and ID administration were approximately five and threefold lower, respectively. The bioavailability (F), based on AUC_(0−∞)_, of propofol was 30% for oral administration and 37% for ID administration. The liberation and appearance of propofol from fospropofol in systemic circulation was rapid following ID and PO administrations with T_max_ of 0.17 and 0.33 h, respectively. The t_1/2_ and T_max_ for propofol tended to be longer after PO administration than it was following ID and IV administrations (Figure 4b).

**Figure 5:**
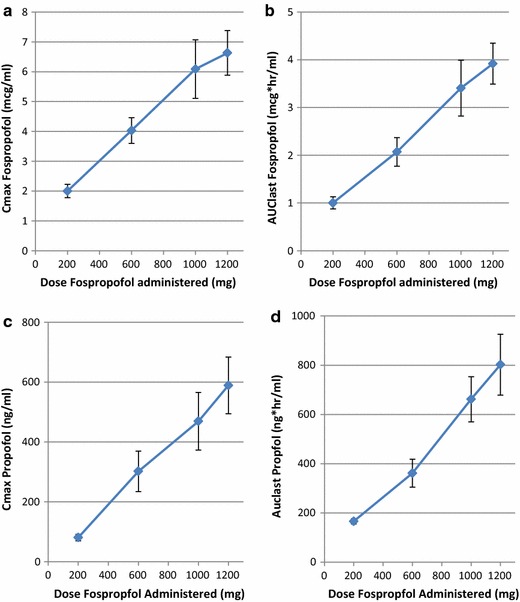
Pharmacokinetic parameters of AUC_last_ and C_max_ for fospropofol (**b **and** a**) and propofol (**d** and **c**) following oral administration of placebo or fospropofol at 200, 400, 600, 1,000 and 1,200 mg in human subjects (n = 10). Each subject received each of the doses. In general, dose proportional plasma concentrations of both fospropofol and propofol were observed. Data shown as mean ± SEM.

*Study 2* In this oral dose escalation study, fospropofol and propofol exposure showed dose related increase following PO doses of fospropofol in capsule form (Figure 5a–d).

### Safety and tolerability of PO and ID fospropofol in human

*Study 1* Seventy-three treatment emergent adverse events (TEAEs) were reported in 7 of 7 subjects (100%), (and are detailed in Additional file 2: Table S1). Seventy of the reported TEAEs were considered to be possibly or probably related to study drug. The most frequently reported TEAEs were somnolence [11 events in 7/7 subjects (100%)], paresthesia [10 events in 6/7 subjects (86%)], speech disorder [6 events in 6/7 subjects (86%)], and burning sensation [6 events in 3/7 subjects (43%)]. Two subjects (29%), one in the PO group and one in the IV group, reported one TEAE each of euphoria. Both events were mild, considered related to study medication and resolved after 17 and 34 min, respectively. No subject experienced a serious adverse event (SAE) and no subject discontinued from the study for any reason. These are detailed in Additional file 2: Table S1.

There was a marked difference in the number of treatment-related TEAEs reported among the different routes of administration. When fospropofol was administered IV, 7 of 7 (100%) subjects reported 56 treatment-related TEAEs. When fospropofol was administered either PO or ID, 6 of 7 (86%) subjects in each group reported 8 and 9 treatment-related TEAEs, respectively. No severe or serious TEAEs were reported during this study. There was no death or study discontinuation because of an AE. All but one TEAE (rash, which resolved without treatment) resolved without sequelae within 1 h of dosing. The Investigator considered all TEAEs mild. No clinically-relevant abnormalities were found with regard to clinical laboratory results, vital signs, ECG, or physical examination.

The Modified OAA/S scale was used to assess subjects’ level of sedation. The lowest observed Modified OAA/S score during this study was 4 (responded lethargically to name spoken in normal tone). Three of 7 (43%) and 4 of 7 (57%) subjects in the ID and IV groups respectively, had a Modified OAA/S score of 4 at some time following drug administration. All other subjects in those treatment groups and all subjects in the oral treatment group responded readily to their name spoken in normal tone (Modified OAA/S score of 5) at all times. All subjects had Modified OAA/S scores of 5 by 1.5 h postdose. This observation of similar sedation levels produced by IV and ID administration compared to PO administration are generally consistent with the pharmacokinetic measurements that suggested somewhat higher duodenal than oral bioavailability.

*Study 2* The patient incidence of TEAEs by treatment was: 40, 80, 90, 80, and 90% for placebo, 200, 600, 1,000, and 1,200 mg, respectively. (These are detailed in Additional file 2: Table S2). Somnolence was reported in 0, 40, 50, 40, and 80% of subjects in the placebo, 200, 600, 1,000, and 1,200 mg groups, respectively. Following somnolence in rate of occurrence were paresthesia (60%), nausea (50%), and phlebitis superficial (50%). Most of the TEAEs were mild or moderate in severity and resolved without intervention. Two subjects (1 in the 1,000 mg treatment group and 1 in the 1,200 mg group) experienced somnolence that was considered severe by the Investigator. Only one TEAE (erythema in the placebo group, considered not related to study drug) required treatment and resolved before the end of the study. Euphoric mood was reported as a TEAE in three subjects during this study; one each in the placebo, 600, and 1,200 mg groups. There were no changes in laboratory values, vital signs, ECGs, or physical examinations that were considered clinically relevant by the Investigator during this study. No subject experienced a SAE and no subject discontinued from the study for any reason.

At most time points ≥80% of subjects in each of the treatment groups responded readily to their names spoken in a normal tone (Modified OAA/S scores of 5). However, at the 1.5-h time point in the 1,200 mg treatment group, 40% of subjects had a Modified OAA/S score of 4 (responded lethargically to their names spoken in a normal tone). The lowest Modified OAA/S scores (score of 3; responded only after name was called loudly and/or repeatedly) were recorded by the same subject (Subject 003, following treatment with 1,000 mg), at 1 and 1½ h after treatment with fospropofol disodium.

DSST (digital symbol substitution test) performance decreased in a dose dependent manner. The maximal DSST changes from baseline for all fospropofol disodium treatment groups were recorded at the 1-h time point. At 1 h post-treatment, mean changes were 6, −5, −11, and −13 for the 200, 600, 1,000, and 1,200 mg groups, respectively. However BIS (bispectral index score) was not affected by fospropofol administration at any dose level where mean BIS scores were ≥90% at all the time points for all subjects following all treatments. Ranges were 67–98%, 80–98%, 71–98%, 70–98%, and 70–98% for the placebo, 200, 600, 1,000, and 1,200 mg groups, respectively.

## Discussion

These results demonstrate for the first time that the water soluble prodrug fospropofol can be used to provide oral bioavailability of propofol in both rat and human. Previous reports concluded that propofol itself has little or no oral bioavailability, presumably due to first pass hepatic metabolism based upon liver extraction of 80% after intravenous administration of lipid emulsion in animals [22–24] and in human [25, 26]. In contrast, we observed that PO and ID fospropofol administration can achieve bioavailability of 30% or more. Interestingly, while propofol availability derived from the prodrug is appreciable; the bioavailability of the prodrug itself is low, suggesting that propofol is liberated from prodrug before entering the central compartment. This suggests that prodrug delivery allows propofol in the portal circulation to avoid first pass metabolism by the liver. GI administration resulted in a delayed, lower C_max_ compared to intravenous propofol or fospropofol but was able to achieve plasma concentrations associated with sedation or analgesia for an hour or more after a single administration. These results suggest for the first time that oral administration of its prodrug may allow safe and practical administration of propofol at subhypnotic exposures for use in treatment of migraine, anxiety or other disease states.

We found that bioavailability and sedative effect varied by dose and location of administration in the GI tract of the rat. On a dose basis, fospropofol induced full sedation (an average score of 3.5 or higher, according to the 0–4 rating scale described in “Methods”, for at least two consecutive recording times) when administered IV at a dose of 40 mg/kg. PO administration produced full sedation scores at 300–400 mg/kg, but only 50 mg/kg was required when administered ID. The time to reach effect and duration was similar through IV or ID routes, but slightly slower in onset and more prolonged following the larger doses required to reach sedation after oral administration, although comparable doses were not administered by both ID and PO routes. This suggests that, at least in rats, either the mechanism by which propofol liberated from fospropofol is dependent in part on the properties of the GI tract or that the conditions in the stomach may partially hydrolyze the prodrug to propofol in the lumen, rendering the propofol unavailable for absorption.

Measurement of plasma propofol in rats confirmed the greater bioavailability of ID compared to PO administration. Interestingly, bioavailability of fospropofol was consistently low when administered at either site in the GI tract, suggesting that the prodrug is hydrolyzed to release propofol prior to reaching the central compartment. Relative propofol bioavailability rose with PO or ID doses, reaching 70% or more at the highest doses administered. The difference in dose required by the PO route to achieve sedation appeared to be due largely to the lower C_max_ even as propofol bioavailability increased on an AUC basis. The higher C_max_ for the ID route corresponded to a lower required dose for full sedation.

While food effect was not formally studied, it should be noted that all pharmacokinetic and sedative animal studies were conducted in the fasted state whereas the neuropathic pain studies were undertaken in rats with free access to food. It is likely that fospropofol would be deemed a class 1 drug under the Biopharmaceutics Classification System, due its high permeability and solubility, so that a food effect would not be expected. Future clinical development of fospropofol through the oral route, however, would require a specific study of food effects.

The clinical observations described provide supporting evidence that fospropofol is bioavailable through the GI tract as well. Dose dependent sedative effects were observed in volunteers administered fospropofol orally by capsule in a dose response study.

The human pharmacokinetic data differed somewhat from rat, suggesting more equal bioavailability from PO and ID administration routes and less dose dependence of bioavailability. However there was lower bioavailability from capsules and significant variability in C_max_, suggesting that a food effect may be likely and that a formulation providing predictable blood levels would be desirable, especially given the potentially narrow therapeutic window of propofol.

The bioavailability of fosproprofol through the GI tract is in marked contrast to the lack of bioavailability reported for propofol administered orally to rats and man in an oil/water emulsion formulation, rectally as an oil or in its pure form in soft gelatin capsules [22–24]. This has been explained in part by the high extraction of propofol by the liver in animals and man after intravenous administration in an emulsion. Importantly, it has been shown that propofol can be absorbed buccally when administered in a semifluorinated alkane based formulation [39] supporting the notion that liver metabolism likely limits oral bioavailability of propofol as oil in emulsion. Raoof et al. [23] studied the relative contribution of intestinal mucosa, liver and lung to in vivo disposition in the rat. In this study AUC’s of propofol were estimated and fractions of the administered dose escaping first pass metabolism by the gut wall (fa), liver (fh) and lung (fl) were calculated using propofol concentration following intra-arterial, intravenous, hepatic portal and oral routes of propofol administration. It was observed that the intestinal mucosa is the main site of first pass metabolism following oral administration of propofol in the rat. The liver and lung contribute much less compared to intestinal mucosa. Intestinal metabolism could therefore also contribute to the systemic clearance of propofol. Due to first pass effect the observed bioavailability of propofol was low (10%).

The oral bioavailability of any drug may be limited by its aqueous solubility, low permeability, propensity to be an efflux substrate, and rapid and extensive hepatic metabolism and biliary excretion. Raoof et al. [23] also reported that propofol is a highly permeable drug (evaluated using Caco-2 cell monolayers) and known to be a poorly soluble drug. Therefore it can be classified as a biopharmaceutical class (BCS) class II drug. The oral bioavailability of this class of compounds is limited by solubility and not permeability. Our study data is consistent with fact that the propofol bioavailability is markedly higher following PO and ID administration of prodrug fospropofol due to its solubility. The absorbed fospropofol rapidly converts to propofol by alkaline phosphatase present in different organs including blood and liver. The low levels of fospropofol following PO administration further support this. Once propofol is in systemic circulation then its disposition is similar to that following IV administration.

Our data suggests that the prodrug is hydrolyzed at the gut wall or in the liver, liberating propofol into the central compartment since the prodrug is not seen at appreciable concentrations in the central compartment after oral administration. We assume that hydrolysis does not occur in the lumen of the gut, since that would result in very low bioavailability like that of propofol. We think it unlikely, based on the physiochemical properties of the phosphono-*O*-methyl prodrug, that fospropofol is absorbed across the gut wall to be hydrolyzed in the portal vein or liver. Therefore it is most likely that hydrolysis takes place at the gut wall and propofol is delivered as free propofol into the portal vein.

This leaves the question of why propofol liberated from the prodrug at the gut wall is handled differently from propofol in emulsion. Propofol is known to be highly bound to serum proteins, particularly albumin [40]. It is therefore likely that propofol is cleaved at the gut wall and then rapidly diffuses to bind to plasma proteins. As a highly bound drug, extraction by the liver may be limited. The dose dependent increase in bioavailability is consistent with observations that free propofol fraction is higher at low plasma propofol concentrations, thus enhancing clearance at low doses compared to higher doses where the free fraction is lower.

We hypothesize that the low bioavailability of propofol administered orally or rectally as an emulsion or pure oil is due to propofol binding to plasma lipoproteins that facilitate its active uptake by the liver and subsequent metabolism. It has been shown that formulation has a significant effect on propofol distribution after intravenous administration with the emulsion formulation enhancing rapid brain effect and preventing pulmonary distribution [39, 41, 42]. While propofol distribution and metabolism has been extensively studied in many species and in clinical settings, major gaps remain in our understanding of its metabolism. For example, despite the high extraction ratio of propofol by the liver, during liver transplant only minor changes in propofol plasma concentration are observed, even as this major metabolic site is removed during the anhepatic phase. Physiological based modeling efforts have been attempted based on blood flow and tissue metabolism, but fail to predict clinical data and require adjustments to match clinical observations. It may be that intravenous infusion of propofol as emulsion is also influenced by factors such as lipoprotein vs albumin binding that constitute distinct and time varying pools of propofol that are not present when propofol is liberated from a water soluble prodrug. These considerations suggest that more detailed metabolic investigation comparing prodrug to emulsion might prove interesting, including measures of liver extraction of propofol from prodrug compared to emulsion when administered intravenously and through the gastrointestinal tract.

The oral bioavailability was sufficient to show analgesic effects in a rodent neuropathic pain model. Non-sedative doses of 75 or 100 mg/kg were effective after a single dose consistent with achieving plasma concentrations greater than 200 ng/mL. Effects were of short duration, losing activity by 2 h after administration, consistent with the pharmacokinetic profile. These observations are consistent with previous reports of analgesic properties of intrathecal propofol in some acute pain models (e.g. phases 1 and 2 of formalin pain, hotplate and acetic acid writhing), but not in others (tail-flick test) [12, 43], and clinical observations of analgesic properties [11]. The demonstration of pharmacologic activity consistent with plasma concentrations confirms that propofol measured in the plasma is fully biologically active. It should be noted that recent publications have shown that environmental enrichment can reduce pain perception in rats [44, 45]. The animals in this study, however, were not provided environmental enrichment. Future studies should monitor and compare the analgesic efficacy of propofol with and without enriching conditions.

The analgesic activity of propofol is consistent with its effects as a modulator of gamma-aminobutyric acid (GABA A) neurotransmission, in the spinal cord as well as the central nervous system [46]. Specifically, the effects of propofol on GABA A receptor mediated presynaptic inhibition at primary afferent terminals in the human spinal cord [47] are thought to decrease spinal nociception [48]. The reported clinical effects of propofol on migraine and nausea may also be through GABAergic modulatory effects, although the definitive pathways have not been clearly described. Classical benzodiazepines that enhance GABA neurotransmission are not recommended as first line therapy for chronic pain because of CNS side effects and potential worsening of pain syndromes with prolonged use. It is possible however, that propofol’s analgesic effects may be mediated through additional mechanisms such as glutamatergic transmission, sodium channel blockade and NMDA/AMPA receptors [43, 49]. A recent publication [50] suggests that HCN (hyperpolarization activated, cyclic nucleotide regulated) channels may also mediate effects of propofol in neuropathic pain models. Regardless of precise mechanism, the clinical utility of propofol oral administration as the prodrug would require longer clinical trials to ensure durable benefit with good tolerability.

Our results suggest that the ability to deliver propofol through non-intravenous routes could enable therapeutic utility of this broad pharmacology. Clinical experience and a number of trials provide substantial evidence of propofol’s clinical usefulness for treatment of epilepsy, pain, nausea and migraine headache. These studies establish for the first time that, when administered as fospropofol, propofol is orally bioavailable in human volunteers. Finally, in a well validated animal model of neuropathic pain, analgesic effects can be demonstrated by oral administration.

## Conclusions

Propofol is a widely used anesthetic/sedative agent whose diverse pharmacology has shown utility in several clinical conditions including treatment of migraine, nausea, pain and anxiety. However its physical properties, including limited solubility and negligible bioavailability via non-intravenous routes, have impeded its more widespread use. Herein we show that oral administration of the fospropofol prodrug provides appreciable propofol bioavailability in both animal and in human volunteers. Furthermore we demonstrate pharmacological efficacy of oral fospropofol in animal models. These data suggest utility of oral administration of fospropofol for various therapeutic indications previously considered for propofol.

## Additional files

Additional file 1: Figure S1. Absolute latency of ipsilateral paw in CCI rats before and after fospropofol or vehicle treatment. Additional file 2: Tables S1 and S2. Table 1: Frequency table of all emergent adverse events from Study 1 and Table 2: Frequency table of all emergent adverse events from Study 2.

## Abbreviations

IV: intravenous
PO: oral
ID: intraduodenal
ALP: alkaline phosphatase
SOV: sodium orthovanadate
TEAE: treatment emergent adverse event
