# Supplementary material for: Gastrointestinal delivery of propofol from fospropofol: its bioavailability and activity in rodents and human volunteers
Source: J Transl Med. 2015 May 29;13:170. doi: 10.1186/s12967-015-0526-9 (PMC4448313; doi:10.1186/s12967-015-0526-9)
Supplement: Additional file 1: Figure S1. — Absolute latency of ipsilateral paw in CCI rats before and after fospropofol or vehicle treatment. [file 12967_2015_526_MOESM1_ESM.docx]

**Supplementary Figure 1:**

Rats subjected to CCI showed similar withdrawal latency on ligated side prior to fospropofol treatment (pretreatment). Following fospropofol administration, the absolute latency of the ligated side showed a dose dependent increase compared to vehicle treatment (post-treatment).  Testing was performed prior to dosing and 1 hour following oral fospropofol or vehicle treatment.
