# Supplementary material for: Gastrointestinal delivery of propofol from fospropofol: its bioavailability and activity in rodents and human volunteers
Source: J Transl Med. 2015 May 29;13:170. doi: 10.1186/s12967-015-0526-9 (PMC4448313; doi:10.1186/s12967-015-0526-9)
Supplement: Additional file 2: Tables S1 and S2. — Table 1: Frequency table of all emergent adverse events from Study 1 and Table 2: Frequency table of all emergent adverse events from Study 2. [file 12967_2015_526_MOESM2_ESM.docx]

| **Treatment-emergent Adverse Events**  System organ class/preferred Term | Total  (N=7)  E n (%) | T1:PO  (N=7)  E n (%) | T2: ID  (N=7)  E n (%) | R:IV  N=7  E n (%) |
| --- | --- | --- | --- | --- |
| BLOOD AND LYMPHATIC SYSTEM DISORDERS | 1 1 (14%) |  |  | 1 1 (14%) |
| lymph node pain | 1 1 (14%) |  |  | 1 1 (14%) |
| EYE DISORDERS | 5 5 (71%) |  |  | 5 5 (71%) |
| diplopia | 1 1 (14%) |  |  | 1 1 (14%) |
| visual disturbance | 4 4 (57%) |  |  | 4 4 (54%) |
| GASTROINTESTINAL DISORDERS | 6 4 (57%) | 1 1 (14%) | 1 1 (14%) | 4 3 (43%) |
| dry mouth | 1 1 (14%) |  |  | 1 1 (14%) |
| dyspepsia | 1 1 (14%) |  |  | 1 1 (14%) |
| flatulence | 1 1 (14%) |  | 1 1 (14%) |  |
| nausea | 2 1 (14%) | 1 1 (14%) |  | 1 1 (14%) |
| proctalgia | 1 1 (14%) |  |  | 1 1 (14%) |
| GENERAL DISORDERS AND ADMINISTRATION  SITE CONDITIONS | 12 5 (71%) |  | 3 3 (43%) | 9 4 (57%) |
| catheter site pain | 1 1 (14%) |  |  | 1 1 (14%) |
| fatigue | 4 3 (43%) |  | 2 2 (29%) | 2 2 (29%) |
| feeling abnormal | 1 1 (14%) |  |  | 1 1 (14%) |
| feeling cold | 1 1 (14%) |  |  | 1 1 (14%) |
| feeling drunk | 1 1 (14%) |  |  | 1 1 (14%) |
| sluggishness | 3 3 (43%) |  |  | 3 3 (43%) |
| subrapubic pain | 1 1 (14%) |  | 1 1 (14%) |  |
| INFECTIONS AND INFESTATIONS | 1 1 (14%) |  | 1 1 (14%) |  |
| herpes simplex | 1 1 (14%) |  | 1 1 (14%) |  |
| MUSCULOSKELETAL AND CONNECTIVE  TISSUE DISORDERS | 2 2 (29%) |  |  | 2 2 (29%) |
| muscle fatigue | 2 2 (29%) |  |  | 2 2 (29%) |
| NERVOUS SYSTEM DISORDERS | 40 7 (100%) | 6 4 (57%) | 3 3 (43%) | 31 7(100%) |
| burning sensation | 6 3 (43%) |  |  | 6 3 (43%) |
| dizziness | 5 3 (43%) | 1 1 (14%) | 1 1 (14%) | 3 3 (43%) |
| dizziness postural | 1 1 (14%) |  |  | 1 1 (14%) |
| paresthesia | 10 6 (86%) | 1 1 (14%) |  | 9 6 (86%) |
| somnolence | 11 7 (100%) | 4 4 (57%) | 2 2 (29%) | 5 5 (71%) |
| speech disorder | 6 6 (86%) |  |  | 6 6 (86%) |
| tremor | 1 1 (14%) |  |  | 1 1 (14%) |
| PSYCHIATRIC DISORDERS | 3 3 (43%) | 1 1 (14%) |  | 2 2 (29%) |
| disorientation | 1 1 (14%) |  |  | 1 1 (14%) |
| euphoric mood | 2 2 (29%) | 1 1 (14%) |  | 1 1 (14%) |
| REPRODUCTVE SYSTEM AND BREAST DISORDERS | 1 1 (14%) |  |  | 1 1 (14%) |
| genital pruritis male | 1 1 (14%) |  |  | 1 1 (14%) |
| SKIN AND SUBCUTANEOUS TISSUE DISORDERS | 2 2 (29%) |  | 1 1 (14%) | 1 1 (14%) |
| ecchymosis | 1 1 (14%) |  |  | 1 1 (14%) |
| rash | 1 1 (14%) |  | 1 1 (14%) |  |
| TOTAL | 73 7 (100%) | 8 6 (86%) | 9 6 (86%) | 56 7(100%) |

N: number of subjects exposed

E: number of adverse events

n (%): number and percentage of subjects with adverse events

Note: Each occurrence, independent of whether it may be the same adverse event in the same subject during one treatment was counted

**SUPPLEMENTARY TABLE 1: Frequency table of all treatment-emergent adverse events from Study 1**

| **Treatment-emergent Adverse Events**  System organ Class/preferred term | Total  (N=10)  E n (5%) | Placebo  (N=10)  E n (%) | 200mg  Fospropofol  (N=10)  E n (%) | 600 mg  Fospropofol  (N=10)  E n (%) | 1000 mg  Fospropofol  (N=10)  E n (%) | 1200 mg  Fospropofol  (N=10)  E n (%) |
| --- | --- | --- | --- | --- | --- | --- |
| CARDIAC DISORDERS | 1 1 (10%) |  |  |  | 1 1 (10%) |  |
| palpitations | 1 1 (10%) |  |  |  | 1 1 (10%) |  |
| EYE DISORDERS | 2 2 (20%) | 1 1 (10%) | 1 1 (10%) |  |  |  |
| eye irritation | 1 1 (10%) |  | 1 1 (10%) |  |  |  |
| eye pain | 1 1 (10%) | 1 1 (10%) |  |  |  |  |
| GASTROINTESTINAL DISORDERS | 13 8 (80%) |  | 2 1 (10%) | 4 3 (30%) | 4 4 (40%) | 3 3 (30%) |
| abdominal pain | 2 2 (20%) |  | 1 1 (10%) | 1 1 (10%) |  |  |
| bowel sounds abnormal | 1 1 (10%) |  |  | 1 1 (10%) |  |  |
| defecation urgency | 1 1 (10%) |  |  |  |  | 1 1 (10%) |
| dysphagia | 1 1 (10%) |  |  |  | 1 1 (10%) |  |
| nausea | 7 5 (50%) |  | 1 1 (10%) | 1 1 (10%) | 3 3 (30%) | 2 2 (20%) |
| proctalgia | 1 1 (10%) |  |  | 1 1 (10%) |  |  |
| GENERAL DISORDERS AND ADMINISTRATION SITE CONDITIONS | 15 4 (40%) |  | 1 1 (10%) | 5 4 (40%) | 3 3 (30%) | 6 3 (30%) |
| fatigue | 4 2 (20%) |  |  | 2 2 (20%) | 1 1 (10%) | 1 1 (10%) |
| feeling hot | 10 4 (40%) |  | 1 1 (10%) | 3 3 (30%) | 2 2 (20%) | 4 3 (30%) |
| malaise | 1 1 (10%) |  |  |  |  |  |
| INFECTIONS AND INFESTATIONS | 2 2 (20%) | 1 1 (10%) | 1 1 (10%) |  |  | 1 1 (14%) |
| rhinitis | 2 2 (20%) | 1 1 (10%) | 1 1 (10%) |  |  |  |
| MUSCULOSKELETAL AND CONNECTIVE DISORDERS | 1 1 (20%) |  | 1 1 (10%) |  |  |  |
| sensation of heaviness | 1 1 (10%) |  | 1 1 (10%) |  |  |  |
| NERVOUS SYSTEM DISORDERS | 53 10 (100%) |  | 10 7 (70%) | 16 7 (70%) | 14 7 (70%) | 13 9 (90%) |
| dizziness | 2 2 (20%) |  |  | 2 2 (20%) |  |  |
| dysgeusia | 1 1 (10%) |  |  | 1 1 (10%) |  |  |
| headache | 9 3 (30%) |  |  | 3 2 (20%) | 2 2 (20%) | 1 1 (10%) |
| paraesthesia | 18 6 (60%) |  | 3 2 (20%) | 5 5 (50%) | 7 6 (60%) | 3 3 (30%) |
| paraesthesia oral | 2 1 (10%) |  | 3 3 (30%) |  | 1 1 (10%) | 1 1 (10%) |
| somnolence | 21 10 (100%) |  | 4 4 (10%) | 5 5 (50%) | 4 4 (40%) | 8 8 (80%) |
| PSYCHIATRIC DISORDERS | 3 3 (30%) | 1 1 (10%) |  | 1 1 (10%) |  | 1 1 (10%) |
| euphoric mood | 3 3 (30%) | 1 1 (10%) |  | 1 1 (10%) |  | 1 1 (10%) |
| REPRODUCTIVE SYSTEM AND BREAST DISORDERS | 3 1 (10%) |  |  | 1 1 (10%) | 1 1 (10%) | 1 1 (10%) |
| perineal pain | 3 1 (10%) |  |  | 1 1 (10%) | 1 1 (10%) | 1 1 (10%) |
| RESPIRATORY THORACIC AND MEDIASTINAL DISORDERS | 5 4 (40%) | 1 1 (10%) | 1 1 (10%) | 1 1 (10%) | 1 1 (10%) | 1 1 (10%) |
| dyspnoea | 1 1 (10%) |  |  |  |  | 1 1 (10%) |
| pharyngolaryngeal pain | 3 3 (30%) | 1 1 (10%) | 1 1 (10%) | 1 1 (10%) |  |  |
| throat irritation | 1 1 (10%) |  |  |  | 1 1 (10%) |  |
| SKIN AND SUBCUTANEOUS TISSUE DISORDERS | 1 1 (10%) | 1 1 (10%) |  |  |  |  |
| erythema | 1 1 (10%) | 1 1 (10%) |  |  |  |  |
| VASCULAR DISORDERS | 9 6 (60%) | 2 2 (20%) | 1 1 (10%) | 1 1 (10%) | 4 3 (30%) | 1 1 (10%) |
| phlebitis superficial | 6 5 (50%) | 1 1 (10%) |  | 1 1 (10%) | 4 3 (30%) |  |
| vein pain | 3 2 (20%) | 1 1 (10%) | 1 1 (10%) |  |  | 1 1 (10%) |
| TOTAL | 108 10 (100%) | 7 4 (40%) | 18 8 (80%) | 29 9 (90%) | 28 8 (80%) | 26 9 (90%) |

N: number of subjects exposed

E: number of adverse events

n (%): number and percentage of subjects with adverse events

Note: Each occurrence, independent of whether it may be the same adverse event in the same subject during one treatment was counted

**SUPPLEMENTARY TABLE 2: Frequency table of all treatment – emergent adverse events in Study 2, by system organ class and preferred terminology (number of events and number of subjects).**
